# Pennsylvania State Veterinary Medical Association

**Published:** 1892-10

**Authors:** Robt. Gladfelter

**Affiliations:** Rec. Secretary


*Pennsylvania State Veterinary Medical Association.*—The semi-annual meeting of the Pennsylvania State Veterinary Medical Association assembled in the parlors of the Allen House, and was called to order by President W. Horace Hoskins at 10.30 A. M.

At roll call the following members responded : Drs. Collins, Deimer, Dreibelbis, J. C. Michener, Gladfelter, Hart, Hoskins, Keelor, Keil, Kooker, J. C. Foelker, Pearson, Radley, Thos. B. Rayner, Jas. B. Rayner, Ridge, Rohn, Schaufler, Smith and Timberman.

Those present as visitors were : Drs. A. R. May, Boiling Springs, Pa.; W. G. Benner, Doylestown, Pa.; S. J Foelker, Allentown, Pa.; D. Waugh, Pittsburg, Pa.; Heckenberger, Sr., Catasauqua, Pa.; Heckenberger, Jr., Catasauqua, Pa.; H. P. Eves, Wilmington, Del.; B. F. King, Little Silver, N. J.; V. B. Weaver, Centre Valley, Pa.

The minutes of the preceding meeting were read, and on motion adopted.

The new members proposed were : Drs. V. B. Weaver, non-graduate, Centre Valley, Pa ; W. L. Hart, Philadelphia, Pa., graduate of the American Veterinary College ; Chas. J. Harrick, M.R.C.V.S., McKeesport, Pa.; Francis S. Allen, Philadelphia. graduate of American Veterinary College ; James C. McNeil, Pittsburg, graduate of the Dept. Veterinary Medicine, University of Pennsylvania ; John Doris, Pittsburg, Pa., graduate of the Ohio Veterinary College ; Geo. C. Blaker, Richboro, graduate of the American Veterinary College ; A. H. Dorney, Easton, Pa., graduate of the Ontario Veterinary College ; W. G. Banner, Doylestown, Pa., graduate of the Ontario Veterinary College ; S. J. Foelker, Allentown, Pa., graduate of the Ontario Veterinary College ; W. L. Nunan, Lansdown, Pa., graduate of the American Veterinary College ; J. F. Ferley, Philadelphia, graduate of the American Veterinary College ; David Martin, McKeesport, Pa.

A recess was then taken until the Board of Censors examined the applications for membership, and also considered some recommendations to dispose of the charges which had previously been preferred against Dr. Gottleib Meyers, from Allegheny, who registered as a graduate from a veterinary College in Munich, Bavaria, but upon examination it was found that he held a certificate of attendance at an agricultural school, the same not being a veterinary school nor had the power of conferring the degree. The President also requested the Board to devise and recommend some means by which the treasury of the association could be replenished, the surplus having been drained by expenses for printing, postage, etc. The Board thereupon convened ; when through with their deliberations reported favorably, recommending all the applications presented but that of Dr. David Martin, who was absent and did not appear before the Board. His petition was then laid over until the March meeting. The Board further recommends that Dr. Gottleib Meyers be expelled from the association, that an assessment of two dollars be levied upon each member of the association in order that the expenses already incurred might be liquidated.

On motion the report of the Board of Trustees was received and their various recommendations approved.

The next in order was the Secretary's report, which gave a brief summary of the work performed during the past six months, summarizing the registrations in many of the counties during the past six months. He made an earnest appeal for greater individual work on the part of the members.

The meeting then adjourned for lunch, and re-convening at 2.20 P.M., the President referred feelingly to the death of Dr. J. B. Wetz, and asked for the report of the Committee on Resolutions relating to the same. Dr. W. S. Kooker, chairman, rendered his report.

Dr. A. Diemer, member of the Committee on Intelligence and Education, in absence of the chairman, Dr. Weber, made an earnest appeal for stronger fellowship and greater unity of purpose among the members of the profession.

Dr. W. S. Kooker, Chairman of the Committee on Legislation, reported but few violations of the act during the past six months. He advised the prosecution of Drs. R. M. Miller, of Carlisle, and Marcus Porter, Pittsburg, and that an injunction be asked for against the Prothonotary of Allegheny County, on the grounds of violating the intent of the act, in continuing to register non-graduates He reported also the case of a non-graduate in Lancaster County, who was appealing for the privilege of registering, on the grounds that the act was unconstitutional.

Dr. S. J. J. Harger, Chairman Committee on Sanitary Science and Police, being absent, had forwarded his report, and the President asked Secretary Ridge to read the same.

The sense of the association was expressed favorably in regard to propose sanitary laws.

The following amendment was adopted : That the annual meeting of this association shall be two days in order to more thoroughly complete our work.

Papers being in order, Dr. Leonard Pearson gave a very complete summary of the views of all the leading investigators on the subject of Tuberculosis in Cattle, and particularly referring to the value of Tuberculin as a diagnostic agent. He reported many interesting and favorable results in his own experience. Dr. Jas. A. Waugh being absent he had sent his brother, Mr. D. Waugh, to demonstrate his method of castrating Cryptorchids.

The following delegates were appointed to the United States Veterinary Medical Meeting, at Boston, on the 20th: Drs. J. C. Welsar, J. Curtis Michener, and Jno. R. Hart. Dr. Michener declining, Dr. Geo. B. Rayner was appointed to fill the vacancy.

To the Veterinary Medical Association of New Jersey, Drs. W. S. Kooker, W. H. Ridge, and Leonard Pearson were appointed delegates for the next six months. After which the meeting adjourned. ROBT. GLADFELTER, V. S., *Rec. Secretary.*

